# 4,4′-[2,5-Bis(dodec­yloxy)-*p*-phenyl­ene]bis­(2-methyl­but-3-yn-2-ol)

**DOI:** 10.1107/S1600536810019227

**Published:** 2010-06-05

**Authors:** Xiao-wei Zhang, Zhong-wei Gu

**Affiliations:** a College of Chemistry, Sichuan University, Chengdu 610064, People’s Republic of China

## Abstract

In the title compound, C_40_H_66_O_4_, the C and O atoms of the propinyl and dodecoxyl substituents are nearly coplanar with the benzene ring, 1.735 (6), 8.804 (1), 8.786 (1) and 9.577 (3)°, respectively. In the crystal, mol­ecules are connected by inter­molecular O—H⋯O hydrogen bonds.

## Related literature

The title compound is an important inter­mediate for the preparation of π-conjugated polymers and supra­molecular architectures, see Fang *et al.* (2006 ▶); Chou *et al.* (2010 ▶); Mahesh *et al.* (2009 ▶). For background to polyaryl­eneethynylenes (PAEs) and their properties and applications, see: Bunz (2000 ▶, 2005 ▶); Cheng & Luh (2004 ▶); Zhan *et al.* (2001 ▶).
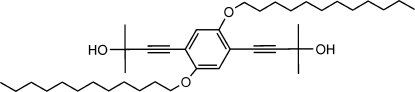


## Experimental

### 

#### Crystal data

C_40_H_66_O_4_
*M* *_r_* = 610.93
Triclinic, 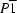

*a* = 9.1325 (9) Å
*b* = 9.707 (1) Å
*c* = 22.9107 (19) Å
α = 85.810 (1)°
β = 88.512 (2)°
γ = 79.373 (1)°
*V* = 1990.7 (3) Å^3^
*Z* = 2
Mo *K*α radiation
μ = 0.06 mm^−1^
*T* = 298 K
0.49 × 0.45 × 0.44 mm

#### Data collection

Bruker SMART CCD area-detector diffractometer
Absorption correction: multi-scan (*SADABS*; Sheldrick, 2002 ▶) *T*_min_ = 0.970, *T*_max_ = 0.973
10561 measured reflections
6920 independent reflections
2398 reflections with *I* > 2σ(*I*)
*R*_int_ = 0.042

#### Refinement

*R*[*F* ^2^ > 2σ(*F* ^2^)] = 0.071
*wR*(*F* ^2^) = 0.166
*S* = 1.07
6920 reflections
404 parameters
H-atom parameters constrained
Δρ_max_ = 0.18 e Å^−3^
Δρ_min_ = −0.21 e Å^−3^

### 

Data collection: *SMART* (Bruker, 1997 ▶); cell refinement: *SAINT* (Bruker, 1997 ▶); data reduction: *SAINT*; program(s) used to solve structure: *SHELXS97* (Sheldrick, 2008 ▶); program(s) used to refine structure: *SHELXL97* (Sheldrick, 2008 ▶); molecular graphics: *SHELXTL*; software used to prepare material for publication: *SHELXTL*.

## Supplementary Material

Crystal structure: contains datablocks I, global. DOI: 10.1107/S1600536810019227/vm2027sup1.cif

Structure factors: contains datablocks I. DOI: 10.1107/S1600536810019227/vm2027Isup2.hkl

Additional supplementary materials:  crystallographic information; 3D view; checkCIF report

## Figures and Tables

**Table 1:** Hydrogen-bond geometry (Å, °)

*D*—H⋯*A*	*D*—H	H⋯*A*	*D*⋯*A*	*D*—H⋯*A*
O3—H3⋯O4^i^	0.82	2.04	2.853 (3)	173
O4—H4⋯O1^i^	0.82	2.35	3.167 (3)	175

Symmetry code: (i) 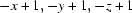.

**Fig. 1.**
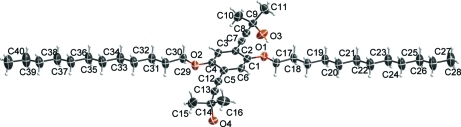
The molecular structure of the title compound showing the atomic numbering and 30% probability displacement ellipsoids.

**Fig. 2.**
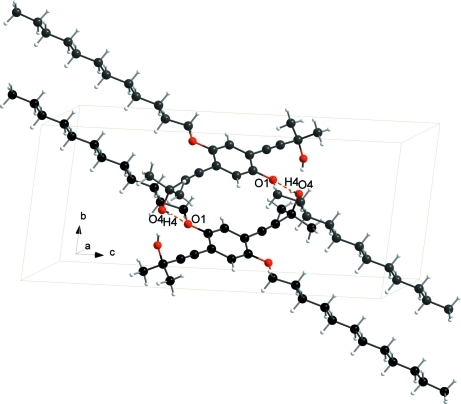
Packinig diagram of the title compound with hydrogen bonds drawn as dashed lines.

## supplementary crystallographic information

### Comment

In the past decade, a family of π-conjugated polymers such as polyaryleneethynylenes (PAEs) have been extensively studied (Bunz, 2000). These compounds usually show functional properities such as photoluminescence (Bunz, 2000) or electroluminescence (Zhan, 2001), and can be used as electronic and photonic devices (Cheng, 2004; Bunz 2005). We have prepared the novel title compound, (I), based on 1,4-dibromo-2,5-bis(dodecyloxy)benzene and 2-methylbut-3-yn-2-ol. It is an important intermediate for the preparation of π-conjugated polymers (Fang *et al.*, 2006) and supramolecular architectures (Chou *et al.*.,2010; Mahesh *et al.*.,2009). In the title compoud, all the C, O atoms of propinyl and dodecoxyl are almost coplanar with the benzene ring. The two dodecyloxy chains show a nice zigzag conformation (Fig. 1). In the crystal, molecules are connected by O—H···O hydrogen bonds and the two benzene rings are parallel in the monoclinic unit cell (Fig. 2).

### Experimental

The title compound was obtained by adding 1,4-dibromo-2,5-bis(dodecyloxy)benzene (10.00 g, 16.54 mmol), triethylamine (80 ml), PdCl_2_(PPh_3_)_2_ (1.16 g, 1.65 mmol), PPh_3_ (2.16 g, 8.25 mmol), 2-methylbut-3-yn-2-ol (3.48 g, 41.35 mmol) and CuI (0.30 g, 1.65 mmol) into a 250 ml three-necked flask. The mixture was heated in an oil bath to reflux for 20 h. After cooling to room temperature, the mixture was filtered and the filtrate was concentrated under reduced pressure. Colourless acicular crystals of the title compound were grown by slow evaporation of an ethanol solution at room temperature. (8.10 g, 80% yield, m.p. 368 K-370 K). Analysis calculated for C_40_H_66_O_4_: C, 78.64; H, 10.89; found: C, 78.60; H, 10.80%.

### Refinement

H atoms bound to C atoms and O atoms were placed in calculated positions and treated as riding on their parent atoms, with C—H = 0.93 Å (aromatic C), C—H = 0.97 Å (methylene C), C—H = 0.96 Å (methyl C), O—H = 0.82 Å and with *U*_iso_(H) = 1.2Ueq(C, O).

### Crystal data

**Table:**

C_40_H_66_O_4_	*Z* = 2
*M**_r_* = 610.93	*F*(000) = 676
Triclinic, *P*1	*D*_x_ = 1.019 Mg m^−^^3^
Hall symbol: -P 1	Mo *K*α radiation, λ = 0.71073 Å
*a* = 9.1325 (9) Å	Cell parameters from 1230 reflections
*b* = 9.707 (1) Å	θ = 2.4–20.8°
*c* = 22.9107 (19) Å	µ = 0.06 mm^−^^1^
α = 85.810 (1)°	*T* = 298 K
β = 88.512 (2)°	Acicular, colorless
γ = 79.373 (1)°	0.49 × 0.45 × 0.44 mm
*V* = 1990.7 (3) Å^3^	

### Data collection

**Table:**

Bruker SMART CCD area-detector diffractometer	6920 independent reflections
Radiation source: fine-focus sealed tube	2398 reflections with *I* > 2σ(*I*)
graphite	*R*_int_ = 0.042
phi and ω scans	θ_max_ = 25.0°, θ_min_ = 1.8°
Absorption correction: multi-scan (*SADABS*; Sheldrick, 2002)	*h* = −10→9
*T*_min_ = 0.970, *T*_max_ = 0.973	*k* = −11→11
10561 measured reflections	*l* = −27→27

### Refinement

**Table:**

Refinement on *F*^2^	Secondary atom site location: difference Fourier map
Least-squares matrix: full	Hydrogen site location: inferred from neighbouring sites
*R*[*F*^2^ > 2σ(*F*^2^)] = 0.071	H-atom parameters constrained
*wR*(*F*^2^) = 0.166	*w* = 1/[σ^2^(*F*_o_^2^) + (0.0353*P*)^2^] where *P* = (*F*_o_^2^ + 2*F*_c_^2^)/3
*S* = 1.07	(Δ/σ)_max_ = 0.001
6920 reflections	Δρ_max_ = 0.18 e Å^−^^3^
404 parameters	Δρ_min_ = −0.21 e Å^−^^3^
0 restraints	Extinction correction: *SHELXL97* (Sheldrick, 2008), Fc^*^=kFc[1+0.001xFc^2^λ^3^/sin(2θ)]^-1/4^
Primary atom site location: structure-invariant direct methods	Extinction coefficient: 0.0060 (6)

### Special details

**Table:**

Geometry. All esds (except the esd in the dihedral angle between two l.s. planes) are estimated using the full covariance matrix. The cell esds are taken into account individually in the estimation of esds in distances, angles and torsion angles; correlations between esds in cell parameters are only used when they are defined by crystal symmetry. An approximate (isotropic) treatment of cell esds is used for estimating esds involving l.s. planes.
Refinement. Refinement of *F*^2^ against ALL reflections. The weighted *R*-factor wR and goodness of fit *S* are based on *F*^2^, conventional *R*-factors *R* are based on *F*, with *F* set to zero for negative *F*^2^. The threshold expression of *F*^2^ > σ(*F*^2^) is used only for calculating *R*-factors(gt) etc. and is not relevant to the choice of reflections for refinement. *R*-factors based on *F*^2^ are statistically about twice as large as those based on *F*, and *R*- factors based on ALL data will be even larger.

### Fractional atomic coordinates and isotropic or equivalent isotropic displacement parameters (Å^2^)

**Table:**

	*x*	*y*	*z*	*U*_iso_*/*U*_eq_	
O1	0.5619 (2)	0.6464 (2)	0.60317 (9)	0.0666 (7)	
O2	0.4531 (2)	0.8819 (2)	0.37806 (9)	0.0714 (7)	
O3	1.0800 (2)	0.7198 (2)	0.62786 (11)	0.0994 (9)	
H3	1.0311	0.6572	0.6339	0.119*	
O4	0.1121 (2)	0.4803 (2)	0.35258 (8)	0.0752 (7)	
H4	0.1964	0.4527	0.3652	0.090*	
C1	0.5291 (3)	0.7034 (3)	0.54744 (14)	0.0534 (8)	
C2	0.6249 (3)	0.7888 (3)	0.52315 (13)	0.0528 (8)	
C3	0.5991 (3)	0.8499 (3)	0.46641 (13)	0.0572 (9)	
H3A	0.6627	0.9073	0.4501	0.069*	
C4	0.4830 (3)	0.8276 (3)	0.43423 (14)	0.0533 (8)	
C5	0.3887 (3)	0.7413 (3)	0.45894 (14)	0.0551 (9)	
C6	0.4118 (3)	0.6820 (3)	0.51588 (13)	0.0584 (9)	
H6	0.3463	0.6270	0.5326	0.070*	
C7	0.7507 (3)	0.8120 (3)	0.55566 (13)	0.0582 (9)	
C8	0.8540 (3)	0.8312 (3)	0.58160 (14)	0.0602 (9)	
C9	0.9813 (4)	0.8515 (4)	0.61596 (15)	0.0635 (9)	
C10	1.0743 (4)	0.9431 (3)	0.58122 (14)	0.0930 (13)	
H10A	1.1587	0.9521	0.6038	0.139*	
H10B	1.0152	1.0344	0.5723	0.139*	
H10C	1.1082	0.9010	0.5455	0.139*	
C11	0.9265 (4)	0.9115 (4)	0.67326 (14)	0.0987 (13)	
H11A	0.8738	0.8475	0.6952	0.148*	
H11B	0.8609	1.0000	0.6656	0.148*	
H11C	1.0100	0.9250	0.6954	0.148*	
C12	0.2687 (3)	0.7079 (3)	0.42565 (13)	0.0601 (9)	
C13	0.1735 (4)	0.6704 (3)	0.40058 (13)	0.0623 (10)	
C14	0.0546 (4)	0.6191 (3)	0.37183 (15)	0.0643 (9)	
C15	0.0049 (4)	0.7068 (4)	0.31614 (15)	0.1171 (16)	
H15A	0.0902	0.7155	0.2918	0.176*	
H15B	−0.0454	0.7984	0.3257	0.176*	
H15C	−0.0618	0.6620	0.2957	0.176*	
C16	−0.0720 (4)	0.6081 (4)	0.41370 (17)	0.1193 (15)	
H16A	−0.1470	0.5705	0.3947	0.179*	
H16B	−0.1139	0.6996	0.4261	0.179*	
H16C	−0.0362	0.5469	0.4472	0.179*	
C17	0.4571 (3)	0.5688 (3)	0.63136 (12)	0.0651 (9)	
H17A	0.4507	0.4879	0.6097	0.078*	
H17B	0.3591	0.6277	0.6324	0.078*	
C18	0.5082 (3)	0.5217 (3)	0.69225 (12)	0.0682 (10)	
H18A	0.5215	0.6026	0.7126	0.082*	
H18B	0.6038	0.4587	0.6907	0.082*	
C19	0.3975 (3)	0.4474 (3)	0.72566 (12)	0.0768 (11)	
H19A	0.3027	0.5114	0.7273	0.092*	
H19B	0.3828	0.3682	0.7044	0.092*	
C20	0.4440 (4)	0.3951 (3)	0.78700 (13)	0.0808 (11)	
H20A	0.4642	0.4735	0.8075	0.097*	
H20B	0.5363	0.3274	0.7851	0.097*	
C21	0.3313 (4)	0.3275 (4)	0.82205 (13)	0.0951 (13)	
H21A	0.3114	0.2490	0.8016	0.114*	
H21B	0.2390	0.3952	0.8237	0.114*	
C22	0.3764 (4)	0.2753 (4)	0.88363 (14)	0.0943 (12)	
H22A	0.4678	0.2065	0.8818	0.113*	
H22B	0.3985	0.3535	0.9036	0.113*	
C23	0.2654 (4)	0.2106 (4)	0.91963 (14)	0.1057 (14)	
H23A	0.1740	0.2794	0.9212	0.127*	
H23B	0.2435	0.1324	0.8995	0.127*	
C24	0.3075 (4)	0.1586 (4)	0.98064 (14)	0.1027 (14)	
H24A	0.3972	0.0876	0.9790	0.123*	
H24B	0.3325	0.2361	1.0004	0.123*	
C25	0.1960 (4)	0.0981 (4)	1.01714 (15)	0.1141 (15)	
H25A	0.1067	0.1695	1.0187	0.137*	
H25B	0.1705	0.0215	0.9970	0.137*	
C26	0.2341 (4)	0.0446 (4)	1.07781 (15)	0.1080 (14)	
H26A	0.3221	−0.0284	1.0762	0.130*	
H26B	0.2618	0.1206	1.0977	0.130*	
C27	0.1228 (5)	−0.0124 (5)	1.11418 (16)	0.1390 (18)	
H27A	0.0949	−0.0879	1.0940	0.167*	
H27B	0.0350	0.0608	1.1156	0.167*	
C28	0.1581 (5)	−0.0666 (5)	1.17449 (17)	0.160 (2)	
H28A	0.2394	−0.1449	1.1744	0.241*	
H28B	0.0724	−0.0964	1.1928	0.241*	
H28C	0.1857	0.0061	1.1958	0.241*	
C29	0.5533 (3)	0.9638 (3)	0.35073 (13)	0.0709 (10)	
H29A	0.6535	0.9090	0.3509	0.085*	
H29B	0.5536	1.0460	0.3724	0.085*	
C30	0.5069 (4)	1.0082 (4)	0.28960 (13)	0.0813 (11)	
H30A	0.4128	1.0735	0.2902	0.098*	
H30B	0.4913	0.9266	0.2702	0.098*	
C31	0.6199 (4)	1.0771 (3)	0.25481 (13)	0.0817 (11)	
H31A	0.6402	1.1545	0.2759	0.098*	
H31B	0.7121	1.0094	0.2527	0.098*	
C32	0.5752 (4)	1.1317 (4)	0.19408 (13)	0.0932 (12)	
H32A	0.5508	1.0549	0.1737	0.112*	
H32B	0.4850	1.2018	0.1965	0.112*	
C33	0.6870 (4)	1.1953 (4)	0.15801 (14)	0.0944 (12)	
H33A	0.7769	1.1250	0.1554	0.113*	
H33B	0.7120	1.2716	0.1786	0.113*	
C34	0.6418 (4)	1.2511 (4)	0.09735 (14)	0.1049 (14)	
H34A	0.6142	1.1752	0.0773	0.126*	
H34B	0.5530	1.3227	0.1002	0.126*	
C35	0.7518 (4)	1.3120 (4)	0.06007 (15)	0.1056 (14)	
H35A	0.7786	1.3882	0.0801	0.127*	
H35B	0.8410	1.2405	0.0578	0.127*	
C36	0.7107 (4)	1.3664 (4)	−0.00021 (15)	0.1068 (14)	
H36A	0.6816	1.2907	−0.0199	0.128*	
H36B	0.6229	1.4393	0.0022	0.128*	
C37	0.8198 (5)	1.4237 (4)	−0.03764 (16)	0.1198 (16)	
H37A	0.9072	1.3503	−0.0401	0.144*	
H37B	0.8495	1.4986	−0.0176	0.144*	
C38	0.7808 (5)	1.4795 (4)	−0.09775 (16)	0.1136 (15)	
H38A	0.7468	1.4060	−0.1173	0.136*	
H38B	0.6963	1.5558	−0.0952	0.136*	
C39	0.8910 (5)	1.5306 (5)	−0.13537 (18)	0.153 (2)	
H39A	0.9739	1.4528	−0.1384	0.184*	
H39B	0.9275	1.6009	−0.1147	0.184*	
C40	0.8588 (5)	1.5906 (5)	−0.19420 (18)	0.167 (2)	
H40A	0.8357	1.5199	−0.2178	0.250*	
H40B	0.9441	1.6251	−0.2105	0.250*	
H40C	0.7752	1.6667	−0.1934	0.250*	

### Atomic displacement parameters (Å^2^)

**Table:**

	*U*^11^	*U*^22^	*U*^33^	*U*^12^	*U*^13^	*U*^23^
O1	0.0536 (14)	0.0819 (16)	0.0665 (15)	−0.0259 (13)	−0.0131 (12)	0.0194 (13)
O2	0.0585 (15)	0.0954 (17)	0.0632 (15)	−0.0284 (14)	−0.0101 (13)	0.0144 (13)
O3	0.0594 (16)	0.0710 (17)	0.169 (2)	−0.0183 (14)	−0.0301 (16)	0.0092 (17)
O4	0.0587 (15)	0.0705 (16)	0.0982 (17)	−0.0143 (13)	−0.0151 (13)	−0.0061 (13)
C1	0.039 (2)	0.056 (2)	0.064 (2)	−0.0101 (18)	−0.0039 (18)	0.0030 (18)
C2	0.0368 (19)	0.053 (2)	0.067 (2)	−0.0070 (17)	−0.0014 (18)	0.0032 (17)
C3	0.040 (2)	0.063 (2)	0.067 (2)	−0.0109 (18)	0.0037 (18)	0.0037 (18)
C4	0.039 (2)	0.056 (2)	0.063 (2)	−0.0056 (18)	−0.0004 (18)	−0.0008 (18)
C5	0.035 (2)	0.057 (2)	0.074 (2)	−0.0103 (18)	−0.0073 (19)	−0.0011 (19)
C6	0.044 (2)	0.063 (2)	0.068 (2)	−0.0136 (18)	0.0001 (19)	0.0074 (19)
C7	0.050 (2)	0.056 (2)	0.070 (2)	−0.0166 (18)	−0.0043 (19)	0.0032 (17)
C8	0.047 (2)	0.061 (2)	0.074 (2)	−0.0148 (18)	−0.0034 (19)	0.0004 (18)
C9	0.048 (2)	0.060 (2)	0.085 (3)	−0.019 (2)	−0.012 (2)	0.003 (2)
C10	0.076 (3)	0.105 (3)	0.108 (3)	−0.049 (3)	−0.011 (2)	0.013 (2)
C11	0.091 (3)	0.139 (4)	0.076 (3)	−0.044 (3)	−0.008 (2)	−0.012 (3)
C12	0.049 (2)	0.062 (2)	0.071 (2)	−0.0150 (19)	−0.0004 (19)	−0.0012 (18)
C13	0.051 (2)	0.063 (2)	0.074 (2)	−0.0145 (19)	−0.003 (2)	0.0005 (19)
C14	0.050 (2)	0.059 (2)	0.085 (3)	−0.010 (2)	−0.009 (2)	−0.008 (2)
C15	0.135 (4)	0.094 (3)	0.127 (3)	−0.036 (3)	−0.082 (3)	0.022 (3)
C16	0.066 (3)	0.148 (4)	0.160 (4)	−0.045 (3)	0.027 (3)	−0.062 (3)
C17	0.049 (2)	0.076 (2)	0.070 (2)	−0.020 (2)	−0.0010 (19)	0.0098 (19)
C18	0.055 (2)	0.088 (3)	0.061 (2)	−0.016 (2)	−0.0048 (19)	0.0107 (19)
C19	0.058 (2)	0.104 (3)	0.068 (2)	−0.022 (2)	0.002 (2)	0.012 (2)
C20	0.071 (3)	0.104 (3)	0.067 (2)	−0.024 (2)	−0.003 (2)	0.016 (2)
C21	0.081 (3)	0.135 (4)	0.071 (3)	−0.033 (3)	0.001 (2)	0.019 (2)
C22	0.090 (3)	0.117 (3)	0.075 (3)	−0.028 (3)	0.001 (2)	0.022 (2)
C23	0.092 (3)	0.148 (4)	0.076 (3)	−0.034 (3)	0.000 (2)	0.025 (3)
C24	0.102 (3)	0.127 (4)	0.076 (3)	−0.025 (3)	0.001 (3)	0.025 (3)
C25	0.106 (3)	0.160 (4)	0.075 (3)	−0.036 (3)	0.004 (3)	0.029 (3)
C26	0.112 (3)	0.132 (4)	0.077 (3)	−0.027 (3)	0.001 (3)	0.026 (3)
C27	0.135 (4)	0.190 (5)	0.088 (3)	−0.041 (4)	0.015 (3)	0.038 (3)
C28	0.182 (5)	0.194 (5)	0.096 (3)	−0.033 (4)	0.010 (3)	0.045 (4)
C29	0.060 (2)	0.083 (3)	0.071 (2)	−0.024 (2)	−0.005 (2)	0.013 (2)
C30	0.070 (3)	0.110 (3)	0.063 (2)	−0.022 (2)	−0.004 (2)	0.016 (2)
C31	0.077 (3)	0.096 (3)	0.071 (2)	−0.023 (2)	−0.004 (2)	0.015 (2)
C32	0.084 (3)	0.128 (3)	0.066 (2)	−0.025 (3)	0.002 (2)	0.013 (2)
C33	0.093 (3)	0.113 (3)	0.077 (3)	−0.027 (3)	0.000 (2)	0.018 (2)
C34	0.097 (3)	0.142 (4)	0.075 (3)	−0.029 (3)	0.002 (3)	0.017 (3)
C35	0.102 (3)	0.132 (4)	0.083 (3)	−0.037 (3)	0.001 (3)	0.028 (3)
C36	0.100 (3)	0.147 (4)	0.073 (3)	−0.031 (3)	0.003 (3)	0.018 (3)
C37	0.124 (4)	0.152 (4)	0.084 (3)	−0.042 (3)	−0.001 (3)	0.034 (3)
C38	0.118 (4)	0.147 (4)	0.076 (3)	−0.034 (3)	0.008 (3)	0.015 (3)
C39	0.149 (5)	0.209 (5)	0.099 (4)	−0.053 (4)	−0.008 (3)	0.053 (4)
C40	0.180 (5)	0.209 (5)	0.104 (4)	−0.037 (5)	0.007 (4)	0.036 (4)

### Geometric parameters (Å, °)

**Table:**

O1—C1	1.372 (3)	C22—H22B	0.9700
O1—C17	1.436 (3)	C23—C24	1.487 (4)
O2—C4	1.369 (3)	C23—H23A	0.9700
O2—C29	1.424 (3)	C23—H23B	0.9700
O3—C9	1.434 (3)	C24—C25	1.478 (4)
O3—H3	0.8200	C24—H24A	0.9700
O4—C14	1.449 (3)	C24—H24B	0.9700
O4—H4	0.8200	C25—C26	1.475 (4)
C1—C6	1.363 (4)	C25—H25A	0.9700
C1—C2	1.392 (3)	C25—H25B	0.9700
C2—C3	1.396 (3)	C26—C27	1.460 (4)
C2—C7	1.446 (4)	C26—H26A	0.9700
C3—C4	1.365 (4)	C26—H26B	0.9700
C3—H3A	0.9300	C27—C28	1.464 (4)
C4—C5	1.391 (3)	C27—H27A	0.9700
C5—C6	1.392 (3)	C27—H27B	0.9700
C5—C12	1.449 (4)	C28—H28A	0.9600
C6—H6	0.9300	C28—H28B	0.9600
C7—C8	1.178 (4)	C28—H28C	0.9600
C8—C9	1.471 (4)	C29—C30	1.484 (3)
C9—C11	1.511 (4)	C29—H29A	0.9700
C9—C10	1.512 (4)	C29—H29B	0.9700
C10—H10A	0.9600	C30—C31	1.511 (3)
C10—H10B	0.9600	C30—H30A	0.9700
C10—H10C	0.9600	C30—H30B	0.9700
C11—H11A	0.9600	C31—C32	1.492 (3)
C11—H11B	0.9600	C31—H31A	0.9700
C11—H11C	0.9600	C31—H31B	0.9700
C12—C13	1.180 (4)	C32—C33	1.490 (4)
C13—C14	1.465 (4)	C32—H32A	0.9700
C14—C16	1.497 (4)	C32—H32B	0.9700
C14—C15	1.512 (4)	C33—C34	1.494 (4)
C15—H15A	0.9600	C33—H33A	0.9700
C15—H15B	0.9600	C33—H33B	0.9700
C15—H15C	0.9600	C34—C35	1.480 (4)
C16—H16A	0.9600	C34—H34A	0.9700
C16—H16B	0.9600	C34—H34B	0.9700
C16—H16C	0.9600	C35—C36	1.475 (4)
C17—C18	1.495 (3)	C35—H35A	0.9700
C17—H17A	0.9700	C35—H35B	0.9700
C17—H17B	0.9700	C36—C37	1.459 (4)
C18—C19	1.509 (3)	C36—H36A	0.9700
C18—H18A	0.9700	C36—H36B	0.9700
C18—H18B	0.9700	C37—C38	1.471 (4)
C19—C20	1.503 (3)	C37—H37A	0.9700
C19—H19A	0.9700	C37—H37B	0.9700
C19—H19B	0.9700	C38—C39	1.442 (4)
C20—C21	1.505 (4)	C38—H38A	0.9700
C20—H20A	0.9700	C38—H38B	0.9700
C20—H20B	0.9700	C39—C40	1.444 (4)
C21—C22	1.505 (4)	C39—H39A	0.9700
C21—H21A	0.9700	C39—H39B	0.9700
C21—H21B	0.9700	C40—H40A	0.9600
C22—C23	1.489 (4)	C40—H40B	0.9600
C22—H22A	0.9700	C40—H40C	0.9600
			
C1—O1—C17	116.8 (2)	H23A—C23—H23B	107.3
C4—O2—C29	117.5 (2)	C25—C24—C23	117.3 (3)
C9—O3—H3	109.5	C25—C24—H24A	108.0
C14—O4—H4	109.5	C23—C24—H24A	108.0
C6—C1—O1	124.6 (3)	C25—C24—H24B	108.0
C6—C1—C2	119.7 (3)	C23—C24—H24B	108.0
O1—C1—C2	115.7 (3)	H24A—C24—H24B	107.2
C1—C2—C3	118.9 (3)	C26—C25—C24	118.9 (3)
C1—C2—C7	120.6 (3)	C26—C25—H25A	107.6
C3—C2—C7	120.4 (3)	C24—C25—H25A	107.6
C4—C3—C2	121.7 (3)	C26—C25—H25B	107.6
C4—C3—H3A	119.1	C24—C25—H25B	107.6
C2—C3—H3A	119.1	H25A—C25—H25B	107.0
C3—C4—O2	124.7 (3)	C27—C26—C25	118.8 (3)
C3—C4—C5	118.7 (3)	C27—C26—H26A	107.6
O2—C4—C5	116.5 (3)	C25—C26—H26A	107.6
C4—C5—C6	120.0 (3)	C27—C26—H26B	107.6
C4—C5—C12	121.0 (3)	C25—C26—H26B	107.6
C6—C5—C12	118.9 (3)	H26A—C26—H26B	107.0
C1—C6—C5	120.8 (3)	C26—C27—C28	119.8 (4)
C1—C6—H6	119.6	C26—C27—H27A	107.4
C5—C6—H6	119.6	C28—C27—H27A	107.4
C8—C7—C2	179.3 (4)	C26—C27—H27B	107.4
C7—C8—C9	177.7 (3)	C28—C27—H27B	107.4
O3—C9—C8	110.1 (3)	H27A—C27—H27B	106.9
O3—C9—C11	109.0 (3)	C27—C28—H28A	109.5
C8—C9—C11	109.9 (3)	C27—C28—H28B	109.5
O3—C9—C10	105.0 (3)	H28A—C28—H28B	109.5
C8—C9—C10	110.8 (3)	C27—C28—H28C	109.5
C11—C9—C10	111.9 (3)	H28A—C28—H28C	109.5
C9—C10—H10A	109.5	H28B—C28—H28C	109.5
C9—C10—H10B	109.5	O2—C29—C30	109.8 (2)
H10A—C10—H10B	109.5	O2—C29—H29A	109.7
C9—C10—H10C	109.5	C30—C29—H29A	109.7
H10A—C10—H10C	109.5	O2—C29—H29B	109.7
H10B—C10—H10C	109.5	C30—C29—H29B	109.7
C9—C11—H11A	109.5	H29A—C29—H29B	108.2
C9—C11—H11B	109.5	C29—C30—C31	113.0 (3)
H11A—C11—H11B	109.5	C29—C30—H30A	109.0
C9—C11—H11C	109.5	C31—C30—H30A	109.0
H11A—C11—H11C	109.5	C29—C30—H30B	109.0
H11B—C11—H11C	109.5	C31—C30—H30B	109.0
C13—C12—C5	174.9 (3)	H30A—C30—H30B	107.8
C12—C13—C14	177.3 (3)	C32—C31—C30	115.3 (3)
O4—C14—C13	109.2 (3)	C32—C31—H31A	108.4
O4—C14—C16	108.1 (3)	C30—C31—H31A	108.4
C13—C14—C16	110.5 (3)	C32—C31—H31B	108.4
O4—C14—C15	104.3 (3)	C30—C31—H31B	108.4
C13—C14—C15	112.0 (3)	H31A—C31—H31B	107.5
C16—C14—C15	112.5 (3)	C33—C32—C31	116.4 (3)
C14—C15—H15A	109.5	C33—C32—H32A	108.2
C14—C15—H15B	109.5	C31—C32—H32A	108.2
H15A—C15—H15B	109.5	C33—C32—H32B	108.2
C14—C15—H15C	109.5	C31—C32—H32B	108.2
H15A—C15—H15C	109.5	H32A—C32—H32B	107.3
H15B—C15—H15C	109.5	C32—C33—C34	116.5 (3)
C14—C16—H16A	109.5	C32—C33—H33A	108.2
C14—C16—H16B	109.5	C34—C33—H33A	108.2
H16A—C16—H16B	109.5	C32—C33—H33B	108.2
C14—C16—H16C	109.5	C34—C33—H33B	108.2
H16A—C16—H16C	109.5	H33A—C33—H33B	107.3
H16B—C16—H16C	109.5	C35—C34—C33	117.7 (3)
O1—C17—C18	108.7 (2)	C35—C34—H34A	107.9
O1—C17—H17A	109.9	C33—C34—H34A	107.9
C18—C17—H17A	109.9	C35—C34—H34B	107.9
O1—C17—H17B	109.9	C33—C34—H34B	107.9
C18—C17—H17B	109.9	H34A—C34—H34B	107.2
H17A—C17—H17B	108.3	C36—C35—C34	118.8 (3)
C17—C18—C19	111.4 (2)	C36—C35—H35A	107.6
C17—C18—H18A	109.4	C34—C35—H35A	107.6
C19—C18—H18A	109.4	C36—C35—H35B	107.6
C17—C18—H18B	109.4	C34—C35—H35B	107.6
C19—C18—H18B	109.4	H35A—C35—H35B	107.1
H18A—C18—H18B	108.0	C37—C36—C35	119.1 (3)
C20—C19—C18	113.9 (3)	C37—C36—H36A	107.5
C20—C19—H19A	108.8	C35—C36—H36A	107.5
C18—C19—H19A	108.8	C37—C36—H36B	107.5
C20—C19—H19B	108.8	C35—C36—H36B	107.5
C18—C19—H19B	108.8	H36A—C36—H36B	107.0
H19A—C19—H19B	107.7	C36—C37—C38	120.0 (4)
C19—C20—C21	114.6 (3)	C36—C37—H37A	107.3
C19—C20—H20A	108.6	C38—C37—H37A	107.3
C21—C20—H20A	108.6	C36—C37—H37B	107.3
C19—C20—H20B	108.6	C38—C37—H37B	107.3
C21—C20—H20B	108.6	H37A—C37—H37B	106.9
H20A—C20—H20B	107.6	C39—C38—C37	119.8 (4)
C20—C21—C22	115.1 (3)	C39—C38—H38A	107.4
C20—C21—H21A	108.5	C37—C38—H38A	107.4
C22—C21—H21A	108.5	C39—C38—H38B	107.4
C20—C21—H21B	108.5	C37—C38—H38B	107.4
C22—C21—H21B	108.5	H38A—C38—H38B	106.9
H21A—C21—H21B	107.5	C38—C39—C40	122.6 (4)
C23—C22—C21	116.1 (3)	C38—C39—H39A	106.7
C23—C22—H22A	108.3	C40—C39—H39A	106.7
C21—C22—H22A	108.3	C38—C39—H39B	106.7
C23—C22—H22B	108.3	C40—C39—H39B	106.7
C21—C22—H22B	108.3	H39A—C39—H39B	106.6
H22A—C22—H22B	107.4	C39—C40—H40A	109.5
C24—C23—C22	117.0 (3)	C39—C40—H40B	109.5
C24—C23—H23A	108.0	H40A—C40—H40B	109.5
C22—C23—H23A	108.0	C39—C40—H40C	109.5
C24—C23—H23B	108.0	H40A—C40—H40C	109.5
C22—C23—H23B	108.0	H40B—C40—H40C	109.5
			
C17—O1—C1—C6	−5.5 (4)	C6—C5—C12—C13	−36 (4)
C17—O1—C1—C2	174.3 (3)	C5—C12—C13—C14	29 (11)
C6—C1—C2—C3	−0.6 (4)	C12—C13—C14—O4	−73 (8)
O1—C1—C2—C3	179.6 (3)	C12—C13—C14—C16	46 (8)
C6—C1—C2—C7	−179.4 (3)	C12—C13—C14—C15	172 (8)
O1—C1—C2—C7	0.7 (4)	C1—O1—C17—C18	−177.2 (2)
C1—C2—C3—C4	−0.2 (4)	O1—C17—C18—C19	176.3 (2)
C7—C2—C3—C4	178.6 (3)	C17—C18—C19—C20	179.0 (3)
C2—C3—C4—O2	−178.6 (3)	C18—C19—C20—C21	176.9 (3)
C2—C3—C4—C5	−0.2 (4)	C19—C20—C21—C22	−179.8 (3)
C29—O2—C4—C3	2.0 (4)	C20—C21—C22—C23	178.8 (3)
C29—O2—C4—C5	−176.4 (3)	C21—C22—C23—C24	−179.8 (3)
C3—C4—C5—C6	1.4 (4)	C22—C23—C24—C25	178.2 (4)
O2—C4—C5—C6	179.9 (3)	C23—C24—C25—C26	179.6 (4)
C3—C4—C5—C12	−176.6 (3)	C24—C25—C26—C27	178.7 (4)
O2—C4—C5—C12	2.0 (4)	C25—C26—C27—C28	179.8 (4)
O1—C1—C6—C5	−178.4 (3)	C4—O2—C29—C30	177.6 (3)
C2—C1—C6—C5	1.8 (5)	O2—C29—C30—C31	−171.4 (3)
C4—C5—C6—C1	−2.2 (4)	C29—C30—C31—C32	−176.5 (3)
C12—C5—C6—C1	175.8 (3)	C30—C31—C32—C33	−177.8 (3)
C1—C2—C7—C8	158 (29)	C31—C32—C33—C34	−179.5 (3)
C3—C2—C7—C8	−20 (29)	C32—C33—C34—C35	−178.6 (3)
C2—C7—C8—C9	−158 (24)	C33—C34—C35—C36	179.4 (4)
C7—C8—C9—O3	62 (9)	C34—C35—C36—C37	−178.6 (4)
C7—C8—C9—C11	−58 (9)	C35—C36—C37—C38	−179.6 (4)
C7—C8—C9—C10	178 (100)	C36—C37—C38—C39	−177.4 (4)
C4—C5—C12—C13	141 (4)	C37—C38—C39—C40	−178.0 (4)

### Hydrogen-bond geometry (Å, °)

**Table:**

*D*—H···*A*	*D*—H	H···*A*	*D*···*A*	*D*—H···*A*
O3—H3···O4^i^	0.82	2.04	2.853 (3)	173
O4—H4···O1^i^	0.82	2.35	3.167 (3)	175

Symmetry codes: (i) −*x*+1, −*y*+1, −*z*+1.

## References

[bb1] Bruker (1997). *SMART*, *SAINT* Bruker AXS Inc., Madison, Wisconsin, USA.

[bb2] Bunz, U. H. F. (2000). *Chem. Rev.* **100**, 1605–1644. 10.1021/cr990257j 11749277

[bb3] Bunz, U. H. F. (2005). *Adv. Polym. Sci.* **177**, 1–52.

[bb4] Cheng, Y. J. & Luh, T. Y. (2004). *J. Organomet. Chem.* **689**, 4137–4148.

[bb5] Chou, C. E., Wang, D., Bagui, M., Hsu, J., Chakraborty, S. & Peng, Z. H. (2010). *J. Lumin.* **130**, 986–994.

[bb6] Fang, Q., Ren, S. J., Xu, B., Du, J. P. & Cao, A. M. (2006). *J. Polym. Sci. Part A Polym. Chem.* **44**, 3797–3806.

[bb7] Mahesh, S., Thirumalai, R., Yagai, S., Kitamura, A. & Ajayaghosh, A. (2009). *Chem. Commun.* pp. 5984–5986. 10.1039/b912392j 19809618

[bb9] Sheldrick, G. M. (2002). *SADABS.* University of Göttingen, Germany.

[bb10] Sheldrick, G. M. (2008). *Acta Cryst.* A**64**, 112–122. 10.1107/S0108767307043930 18156677

[bb11] Zhan, X., Liu, Y., Yu, G., Wu, X., Zhu, D., Sun, R., Wang, D. & Epstein, A. J. (2001). *J. Mater. Chem.* **11**, 1606–1611.

